# ﻿First record of the flat-skulled woolly bat *Kerivouladepressa* and the Indochinese woolly bat *K.dongduongana* (Chiroptera, Vespertilionidae) in China

**DOI:** 10.3897/zookeys.1149.85821

**Published:** 2023-02-22

**Authors:** Xiaoling Liang, Huixian Xie, Yannan Li, Zhenglanyi Huang, Song Li, Yi Wu, Wenhua Yu

**Affiliations:** 1 Key Laboratory of Conservation and Application in Biodiversity of South China, School of Life Sciences, Guangzhou University, Guangzhou 510006, China Guangzhou University Guangzhou China; 2 Kunming Natural History Museum of Zoology, Kunming Institute of Zoology, Chinese Academy of Sciences, Kunming 650223, China Kunming Institute of Zoology, Chinese Academy of Sciences Kunming China

**Keywords:** *COI*, *Cytb*, *Kerivouladongduongana*, morphology, morphometric analyses, new records, phylogenetic inferences, *RAG2*, Yunnan

## Abstract

Recent studies have revealed that the *Kerivouladepressa* complex should be divided into two species, *K.depressa* distributed mainly in Myanmar, Vietnam, Laos and Cambodia, and *K.dongduongana* found only in the Annamite Mountains of Vietnam, Laos and Cambodia. In November 2018 and April 2019, 24 woolly bats were collected by two-band harp traps in Xishuangbanna, Yunnan, China. Based on morphological, morphometric, and phylogenetic (*COI*, *Cytb*, and *RAG2* gene sequences) analyses, these bats were identified as *K.depressa* and *K.dongduongana*, representing two new species records for the country. Including the new records, six *Kerivoula* species have been recorded in China, namely *K.depressa*, *K.dongduongana*, *K.furva*, *K.kachinensis*, *K.picta* and *K.titania*. To facilitate their identification and biological research in the future, we have provided an up-to-date key to all *Kerivoula* species occurring in China.

## ﻿Introduction

The genus *Kerivoula* (Gray, 1842) contains 27 species in the Indomalaya-Australasia and Afrotropic ecozones (Wilson and Mittermeier 2019). Among these, *Kerivoulahardwickii* (sensu stricto) (Horsfield 1824), the most widespread species of the genus has long been treated as a species complex, with up to six recognized subspecies (Ellerman and Morrison-Scott 1951; Hill 1965; Corbet and Hill 1992). Because of the lack of comparative material, Corbet and Hill (1992), Sinha (1999), and Simmons (2005) did not acknowledge any subspecies of *K.hardwickii*. Subsequently, Bates et al. (2007) divided the species into two morphological types, *K.hardwickii* (with domed skull) and *K.depressa* (Miller, 1906) (with flattened skull). However, molecular phylogenies indicate that the taxonomy and systematics of the *K.hardwickii* complex are still ambiguous because of the occurrence of multiple divergent lineages (Francis et al. 2007, 2010; Khan et al. 2010; Douangboubpha et al. 2016; Nguyen et al. 2016). Recent genetic analyses revealed that *K.depressa* can be divided into two distinct clades (Kuo et al. 2017). Tu et al. (2018) described *K.depressa* and *K.dongduongana* (Tu et al. 2018) based on phylogenetic analyses using *COI*, *Cytb*, and *RAG2* sequences from newly obtained specimens and those from previous studies across the Indo-China Peninsula and the Philippines (Hoofer et al. 2003; Stadelmann et al. 2004; Francis et al. 2010; Khan et al. 2010; Wu et al. 2012; Kruskop 2013; Kuo et al. 2014; Kuo et al. 2017). Currently, the *K.hardwickii* complex contains five species: *K.hardwickii* (sensu stricto), *K.kachinensis* (Bates et al., 2004), *K.furva* (Kuo et al., 2017), *K.depressa*, and *K.dongduongana* (Kuo et al. 2017; Tu et al. 2018).

In China, four species of woolly bats from the genus *Kerivoula* have been recorded, including *K.picta* (Pallas, 1767), *K.furva*, *K.kachinensis*, and *K.titania* (Bates et al., 2014) (Wu et al. 2012; Kuo et al. 2017; Tu et al. 2018; Yu et al. 2018; Wilson and Mittermeier 2019; Yu et al. 2022). In November 2018 and April 2019, a series of chiropteran surveys were conducted in the southwestern region of Yunnan Province, and 24 *Kerivoula* individuals were sampled. Based on morphology, morphometric analyses, and phylogenetic inferences using *COI*, *Cytb*, and *RAG2* sequences, they were identified as *K.depressa* and *K.dongduongana*, which represent two new records of *Kerivoula* in China. In this paper, we provided details about these findings, new distribution information and an up-to-date key to identify all *Kerivoula* species occurring in China.

## ﻿Materials and methods

### ﻿Specimen sampling and morphological measurements and analyses

In November 2018 and April 2019, 24 *Kerivoula* bats were collected using two-band harp traps during field surveys in Xishuangbanna Tropical Botanical Garden, Yunnan, China (21°57'17"N, 101°15'26"E and 21°30'58"N, 101°30'38"E). All field survey and sample collection protocols complied with the current laws of Yunnan Province, China. We followed the guidelines of the American Society of Mammalogists (Sikes 2016) for the care and use of animals. All voucher specimens were determined to be adults based on the degree of epiphyseal-diaphyseal fusion (Brunet-Rossinni and Wilkinson 2009). The specimens were preserved in 75% ethanol and deposited at the School of Life Sciences, Guangzhou University, China.

External and skull measurements were taken with a digital caliper to the nearest 0.01 mm following Bates and Harrison (1997) and Bates et al. (2004). Body mass was measured with an electronic scale. Twenty-four adult specimens were examined using six external and eight craniodental measurements following Tu et al. (2018) and Yu et al. (2018), and further morphometric analyses were performed using 20 specimens (Table 1). We conducted a principal component analysis (PCA) and discriminant analysis of principal components (DAPC) of craniodental measurements using the R Core Team (2013) and R add-in packages: psych (Revelle 2013), ade4 (Dray et al. 2007), adegenet (Jombart 2008), FactoMineR (Le et al. 2008), and ggplot2 (Wickham 2016).

## ﻿Phylogenetic inference

We followed the DNA extraction, amplification, and sequencing procedures according to Yu et al. (2022). Three gene sequences from all 24 voucher *Kerivoula* specimens were obtained (GenBank accession numbers: *COI*: OM716930–OM716952; *Cytb*: OM735691–OM735714; *RAG2*: OM735715–OM735736). These sequences were compared with 87 *COI*, 38 *Cytb*, and 28 *RAG2* sequences of the subfamily Kerivoulinae from NCBI nucleotide databases and with the three outgroup species, including *Myotismuricola* (Gray, 1846), *Harpiocephalusharpia* (Temminck, 1840), and *Murinacyclotis* (Dobson, 1872) (Hoofer et al. 2003; Khan et al. 2010; Ruedi et al. 2012). Newly generated and downloaded sequences are detailed in Appendix 1. The final matrices of *COI*, *Cytb*, and *RAG2* contained 110 taxa and 734 bp, 62 taxa and 1220 bp, and 50 taxa and 1267 bp, respectively. We inferred phylogenetic relationships using both Bayesian and maximum likelihood (ML) approaches. Sequences were aligned with MUSCLE (Edgar 2004). Bayesian analyses were performed using MrBayes v.3.2.7 (Ronquist et al. 2012), and the best-fitting models of sequence evolution were selected by MrModeltest v.2.4 (Nylander 2004) using the Akaike information criterion (GTR+I+G for *COI*, HKY+I+G for *Cytb*, HKY+G for *RAG2*). Four independent Markov chains were run, and 10,000,000 Metropolis-coupled Markov Chain Monte Carlo generations with sampling every 1000 generations were set. The first 25% samples were discarded. ML analyses were performed in IQ-TREE (Minh et al. 2020) with the best model setting in ModelFinder (Kalyaanamoorthy et al. 2017) using Bayesian information criterion (TPM2+F+I+G4 for *COI* and *Cytb*, HKY+F+R2 for *RAG2*).

## ﻿Results

### ﻿Morphological examination

**Figure 1.**
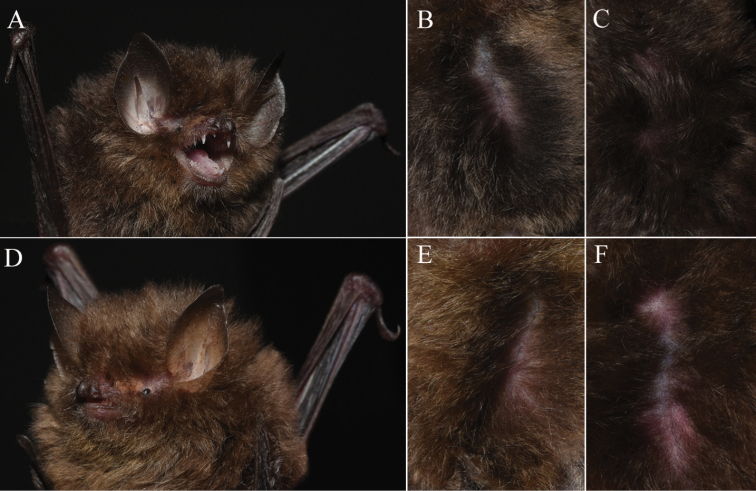
Photographs of *K.depressa* (**A–C** voucher GZHU 19202, male) and *K.dongduongana* (**D–F** voucher GZHU 19198, female) representing their lateral view (**A, D**), ventral pelage (**B, E**) and dorsal pelage (**C, F**).

*Kerivouladepressa* is a moderate-sized species with a forearm (FA) length of 30.75 ± 1.08 mm. Ears are small and rounded, and the posterior margin of the pinnae has a deep, smoothly concave emargination just below the apex. Overall pelage color is buff brown to dark brown. The lower part of ventral hair is dark brown, whereas its tip is light brownish yellow. Dorsal fur is of black base but with dark brown tip (Fig. 1A–C). The domed skull is small, with the greatest length of 13.75 ± 0.17 mm. The mid-portion of the braincase exceeds the frontal region in height. Its lateral profile is flattened from the rostrum to the forehead. A sagittal crest is not evident, and the lambdoid crests are relatively weak. The dental formula is I 2/3, C 1/1, P 3/3, M 3/3. The second upper incisor (*I^3^*) is about half of the first upper incisor (*I^2^*) in height, and the latter is one half the height of the upper canine. The third upper premolar (*P^4^*) is distinctly higher than the anterior two. The third upper molar (*M^3^*) is degenerated. The crown area of the first and second lower molars is approximately equal and slightly larger than the last molar (*M_3_*).

**Figure 2.**
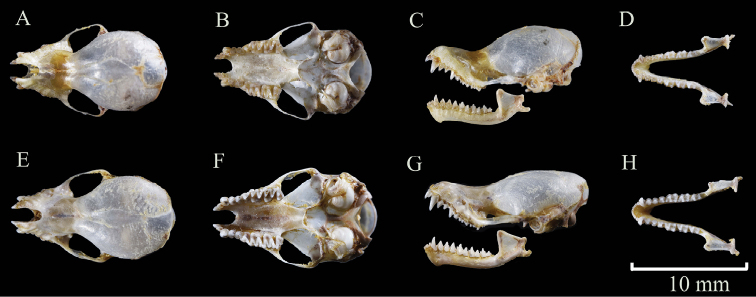
Skull morphology of *K.depressa* (**A–D** voucher GZHU 19222, female) and *K.dongduongana* (**E–H** voucher GZHU 19308, female). Scale bar: 10 mm.

*Kerivouladongduongana*, with FA of 34.02 ± 0.94 mm, is slightly larger than *K.depressa*. Its ears are rounded with a tiny, smooth depression near the tip. *Kerivouladongduongana* is obviously yellower than *K.depressa* in pelage coloration (Fig. 1D–F). The ventral pelage is golden brown, and the base and middle portions are medium brown with golden-brown tips. The dorsal pelage is dark brown with golden-brown tips. Craniodental features are similar to *K.depressa*, but *K.dongduongana* is characterized by a flatter and longer skull (Fig. 2C, G; Table 1).

**Table 1.** External and craniodental measurements (mm) and body mass (g) of studied *Kerivoula* species, and variable loadings on principal components (PCs) and contribution of original variables in discriminant functions (DFs).

Characters	*Kerivouladepressa*		*Kerivouladongduongana*					
	Yunnan, China	Vietnam and Cambodia	Yunnan, China	Vietnam and Cambodia	PC1	PC2	DF1	DF2
	(This study)	Tu et al. 2018	(This study)	Tu et al. 2018				
	Mean ± SD (n) (Range)	Mean ± SD (n) (Range)	Mean ± SD (n) (Range)	Mean ± SD (n) (Range)				
MASS	3.53±0.49 (12) (3.00–4.50)	–	4.05±0.25 (12) (3.50–4.40)	4.50 (1)	–	–	–	–
HB	36.88±2.31 (12) (32.99–39.74)	–	38.43±2.84 (12) (34.40–42.75)	–	–	–	–	–
TL	39.13±1.35 (12) (36.68–41.49)	–	39.97±1.90 (12) (37.20–44.50)	38.00 (1)	–	–	–	–
E	11.55±0.91 (12) (9.96–13.15)	–	12.05±0.89 (12) (10.83–14.04)	–	–	–	–	–
HF	6.98±0.65 (12) (6.09–7.83)	–	7.57±0.48 (12) (7.00–8.43)	–	–	–	–	–
FA	30.75±1.08 (12) (28.82–32.29)	32.08±0.15 (4) (32.00–32.30)	34.02±0.94 (12) (32.56–35.86)	32.00±1.73 (3) (30.00–33.00)	–	–	–	–
TIB	15.95±0.44 (12) (15.38–16.61)	–	16.37±0.58 (11) (15.70–17.44)	18.00 (1)	–	–	–	–
GTL	13.75±0.17 (10) (13.57–14.08)	13.65±0.27 (5) (13.34–13.98)	14.28±0.30 (10) (13.71–14.59)	13.51±0.38 (7) (12.70–13.79)	0.98	–0.14	0.21	0.01
CCL	12.14±0.17 (10) (11.88–12.43)	12.51±0.23 (5) (12.17–12.75)	12.79±0.31 (10) (12.34–13.43)	12.43±0.36 (7) (11.68–12.76)	0.95	–0.24	0.41	0.13
M^3^–M^3^	4.90±0.16 (10) (4.70–5.16)	5.09±0.16 (5) (4.84–5.24)	5.12±0.14 (10) (4.92–5.37)	4.99±0.17 (7) (4.70–5.15)	0.97	–0.02	0.04	0.01
ZB	7.95±0.18 (10) (7.71–8.35)	8.22±0.21 (5) (7.89–8.45)	8.29±0.35 (10) (7.63–8.67)	8.18±0.24 (7) (7.72–8.49)	0.98	–0.10	0.05	–
GBB	6.99±0.11 (10) (6.85–7.15)	7.12±0.05 (5) (7.06–7.19)	6.99±0.11 (10) (6.85–7.15)	7.14±0.20 (7) (6.73–7.31)	–	–	–	–
BH	4.91±0.10 (10) (4.74–5.06)	4.97±0.16 (5) (4.86–5.25)	4.47±0.29 (10) (3.98–4.97)	4.81±0.20 (7) (4.57–5.13)	0.60	0.80	0.29	0.75
C^1^–M^3^	5.11±0.09 (10) (4.94–5.22)	5.37±0.13 (5) (5.17–5.49)	5.23±0.14 (10) (5.00–5.50)	5.25±0.13 (7) (5.06–5.45)	0.97	0.07	–	0.04
ML	9.17±0.28 (10) (8.61–9.56)	9.52±0.21 (5) (9.17–9.75)	9.46±0.29 (10) (8.97–9.88)	9.42±0.26 (7) (9.00–9.78)	0.98	–	0.02	0.06
C_1_–M_3_	5.47±0.14 (10) (5.26–5.65)	5.71±0.15 (5) (5.48–5.86)	5.40±0.22 (10) (4.92–5.71)	5.46±0.15 (7) (5.29–5.76)	0.96	–0.07	0.02	–

### ﻿Multivariate comparison analysis

**Figure 3.**
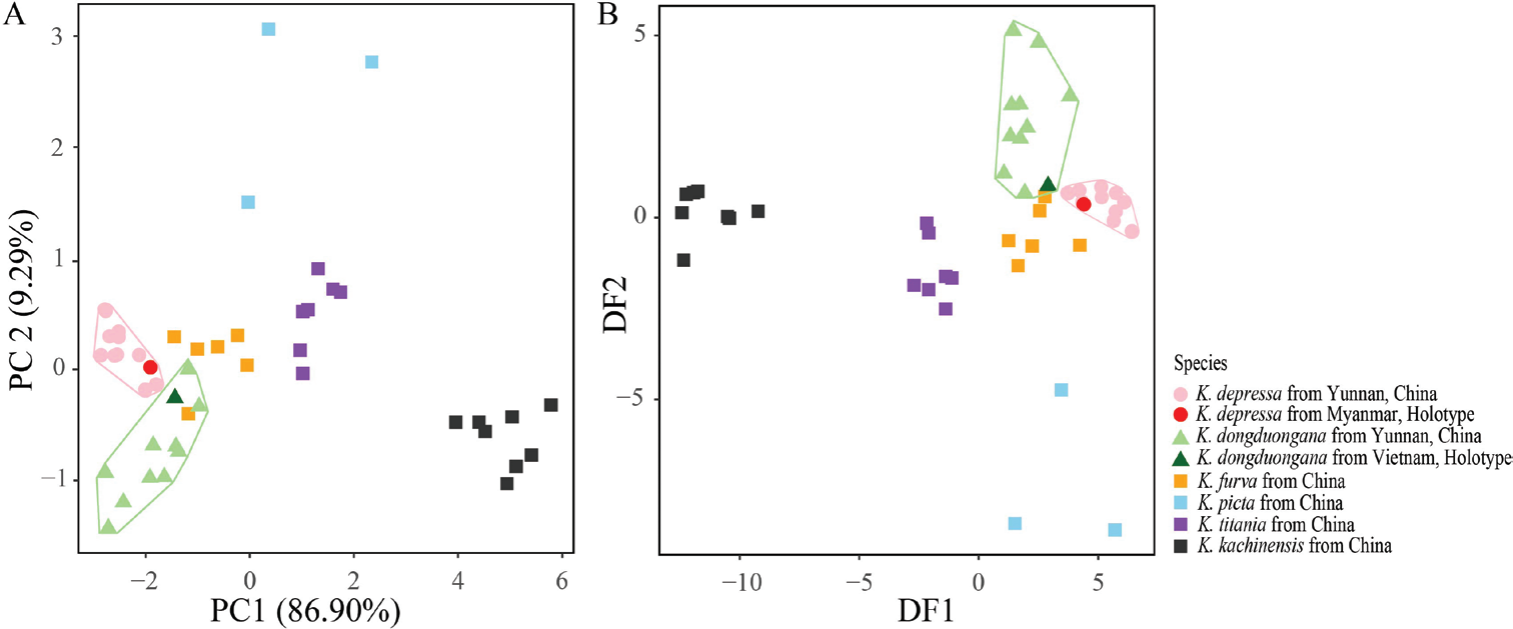
Two-dimensional PCA and DAPC plots of *Kerivoula* species based on nine craniodental measurements **A**PCA plots for *K.depressa*, *K.dongduongana*, *K.furva*, *K.kachinensis*, *K.picta*, and *K.titania* showing projections of individual specimens and variable loadings on the first two principal components **B** projections of 46 specimens and variable loadings on two discriminant functions obtained from external and craniodental measurements.

PCA based on eight craniodental measurements revealed 96.2% of the total variance from the first two principal components (PCs) (86.9% and 9.3% for PC1 and PC2, respectively) in the scatter plot of the six morphological groups (Fig. 3A). For PC1, all measurements had positive loadings (Table 1), reflecting the skull size. Larger bats were characterized by higher PC1 scores; thus, specimens of *K.kachinensis* clustered to the right compared with those of other taxa (Fig. 3A). For PC2, all measurements had low loadings except for the braincase height (BH) (Table 1). Therefore, based on PC2, *K.picta* and *K.titania*, which had a larger BH, were clustered to the top of the plot, whereas *K.dongduongana* and *K.kachinensis* were assigned to the bottom (Fig. 3). For DAPC, we entered the first two PCs from the PCA results and obtained two discriminant functions (DFs) to distinguish among studied *Kerivoula* specimens. PCA and DAPC scatter plots showed that *K.depressa* and *K.dongduongana* specimens formed distinct and separated clusters (pale pink and green triangles in Fig. 3), although some scatter values overlapped with those from *K.furva*. Meanwhile, *K.picta*, *K.titania*, and *K.kachinensis* clustered into three distinguishable groups (Fig. 3).

## ﻿Phylogenetic relationships of *Kerivoula*

**Figure 4.**
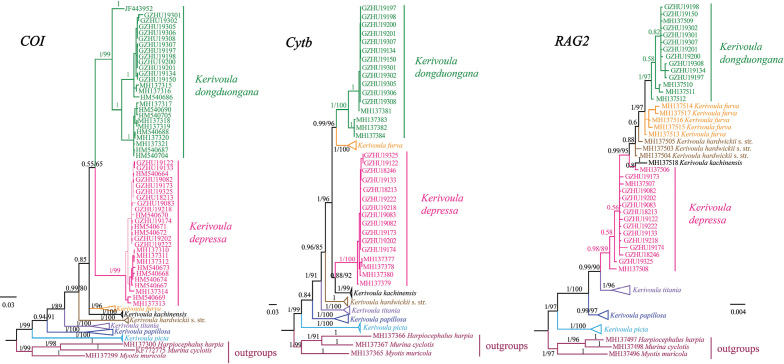
Bayesian and ML trees from analysis of *COI*, *Cytb*, and *RAG2* sequences for the *K.hardwickii* complex. Values on the branches indicate posterior probabilities and bootstrap values, respectively. The terminals *K.picta*, *K.papillosa*, *K.titania*, *K.hardwickii* (sensu stricto), *K.kachinensis*, and *K.furva* each include multiple samples (see Appendix 1).

Bayesian and ML trees using *COI*, *Cytb*, and *RAG2* matrices highly supported monophyly of the genus *Kerivoula* (Fig. 4) [posterior probabilities (PP)/bootstrap values (BS), 1/99 for *COI* and *Cytb*, 1/97 for *RAG2*] and revealed a similar well-supported topology. *Kerivoulapicta* and *K.papillosa* occurred outside of the clade uniting all other examined species within *Kerivoula*. All inferences clustered our sequences with *K.depressa* (PP = 1 in *COI*/*Cytb*; BS: 99 for *COI*, 100 for *Cytb*) and *K.dongduongana* (PP = 1 in *COI*/*Cytb*/*RAG2*; BS: 99 for *COI*, 100 for *Cytb*, 97 for *RAG2*), thus verifying our morphological species identification results (Fig. 4). However, interspecific relationships of *K.depressa*, *K.dongduongana*, *K.kachinensis*, *K.furva*, and *K.hardwickii* (sensu stricto) remain ambiguous and could not be resolved herein.

## ﻿Discussion

The major interspecific phylogenetic relationships of our analyses are comparable with those reported by Kuo et al. (2017) and Tu et al. (2018). Our studies similarly confirmed the monophyly of *K.depressa* and *K.dongduongana*. However, the topology of the phylogenetic tree based on the *RAG2* gene remains unresolved and needs further study. Finally, combining the results of external and craniodental examination and multivariate analyses, 24 specimens were determined as *K.depressa* and *K.dongduongana* (Table 1).

Our discovery of *K.depressa* and *K.dongduongana* in China indicates that six species of *Kerivoula* live in China. According to morphological analyses, *K.picta* is easily distinguished by its unique pelage color pattern and skull shape (Wilson and Mittermeier 2019), whereas *K.kachinensis* is the largest species with a distinctly flattened skull (Bates et al. 2004; Tu et al. 2018; Yu et al. 2022). As for the remaining four similar-sized species, *K.titania* has a distinctly longer tibia and higher braincase than the others (Kuo et al. 2017; Tu et al. 2018). In pelage coloration, *K.furva* has the darkest fur color, varying from black brown to black gray, whereas *K.depressa* and *K.dongduongana* are pale brown. Among the four species, *K.dongduongana* has the shortest BH (Kuo et al. 2017; Tu et al. 2018). A key to the *Kerivoula* species occurring in China is provided in Appendix 2.

Until recently, *Kerivoula* species were considered forest-dependent (Wilson and Mittermeier 2019). They are known in the south of China across Yunnan to Taiwan, and from Hainan to Chongqing. It is worth noting that five of the “Chinese” *Kerivoula* species are found in the southwest region of Yunnan Province, which is often treated as a biodiversity hotspot near Myanmar, Laos, and Vietnam. Its unique terrain, vegetation, and environmental conditions, including a low latitude, warm and tropical forest, and humid micro-climate, appear suitable for inhabitation and colonization (Kruskop 2013; Wilson and Mittermeier 2019; Qian et al. 2020). The high diversity of woolly bats in tropical forest areas may indicate the origin in their diversification progress.

Based on a comparison of the recorded *Kerivoula* diversity from the bordering countries of Myanmar (five species), Laos (seven species), and Vietnam (eight species) (Wilson and Mittermeier 2019), we suggest that there is still a risk of underestimating the diversity of *Kerivoula* in China. More surveys should therefore be conducted, especially on the border/unexplored region and using effective sampling tools such as multi-bank harp traps.

## ﻿Appendix 1

**Table A1.** Sample used in molecular analyses, with GenBank accession numbers for *COI*, *Cytb* and *RAG2* genes provided. Newly generated sequences in this study are shown in bold.

Taxon	Voucher	GenBank accession numbers			Location	Reference
		*COI*	*Cytb*	*RAG2*		
*Kerivouladepressa*	GZHU 18213	**OM716942**	**OM735703**	**OM735730**	Yunnan, China	This study
	GZHU 18246	–	**OM735713**	**OM735736**	Yunnan, China	This study
	GZHU 19082	**OM716943**	**OM735707**	**OM735727**	Yunnan, China	This study
	GZHU 19083	**OM716948**	**OM735704**	**OM735729**	Yunnan, China	This study
	GZHU 19122	**OM716946**	**OM735712**	**OM735731**	Yunnan, China	This study
	GZHU 19133	**OM716947**	**OM735714**	**OM735734**	Yunnan, China	This study
	GZHU 19173	**OM716944**	**OM735708**	**OM735732**	Yunnan, China	This study
	GZHU 19174	**OM716949**	**OM735710**	**OM735725**	Yunnan, China	This study
	GZHU 19202	**OM716950**	**OM735709**	**OM735728**	Yunnan, China	This study
	GZHU 19218	**OM716952**	**OM735705**	**OM735735**	Yunnan, China	This study
	GZHU 19222	**OM716951**	**OM735706**	**OM735733**	Yunnan, China	This study
	GZHU 19325	**OM716945**	**OM735711**	**OM735726**	Yunnan, China	This study
*Kerivouladongduongana*	GZHU 19134	**OM716941**	**OM735697**	**OM735715**	Yunnan, China	This study
	GZHU 19150	**OM716940**	**OM735696**	**OM735720**	Yunnan, China	This study
	GZHU 19197	**OM716936**	**OM735691**	**OM735717**	Yunnan, China	This study
	GZHU 19198	**OM716937**	**OM735692**	**OM735721**	Yunnan, China	This study
	GZHU 19200	**OM716938**	**OM735693**	**OM735722**	Yunnan, China	This study
	GZHU 19201	**OM716939**	**OM735694**	**OM735724**	Yunnan, China	This study
	GZHU 19301	**OM716930**	**OM735698**	**OM735718**	Yunnan, China	This study
	GZHU 19302	**OM716931**	**OM735699**	**OM735719**	Yunnan, China	This study
	GZHU 19305	**OM716932**	**OM735700**	–	Yunnan, China	This study
	GZHU 19306	**OM716933**	**OM735701**	–	Yunnan, China	This study
	GZHU 19307	**OM716935**	**OM735695**	**OM735723**	Yunnan, China	This study
	GZHU 19308	**OM716934**	**OM735702**	**OM735716**	Yunnan, China	This study
*Murinacyclotis*	VN11-1199	KF772775	MH137367	MH137498	Viet Nam	Tu et al. 2018
*Harpiocephalusharpia*	VN11-1288	MH137300	MH137366	MH137497	Viet Nam	Tu et al. 2018
*Myotismuricola*	VN11-1186	MH137299	MH137365	MH137496	Viet Nam	Tu et al. 2018
*Kerivoulapicta*	VN11-1565	MH137303	–	–	Cambodia	Tu et al. 2018
	VN11-1576	MH137304	MH137370	MH137501	Cambodia	Tu et al. 2018
	VN11-1577	MH137305	MH137371	MH137502	Cambodia	Tu et al. 2018
*Kerivoulapapillosa*	20467	MH137301	MH137368	MH137499	Thailand	Tu et al. 2018
	21719	MH137302	MH137369	MH137500	Cambodia	Tu et al. 2018
*K.dongduongana*	ROM 110828	JF443952	–	–	Vietnam	Tu et al. 2018
	CPV10-295	MH137318	–	–	Cambodia	Tu et al. 2018
	CPV10-297	MH137319	MH137382	MH137510	Cambodia	Tu et al. 2018
*K.dongduongana*	ROM MAM 111278	HM540688	–	–	Viet Nam	Francis et al. 2010
	VN11-1158	MH137320	MH137383	MH137511	Vietnam	Tu et al. 2018
	VN11-1178	MH137321	MH137384	MH137512	Vietnam	Tu et al. 2018
	ROM MAM 111298	HM540687	–	–	Viet Nam	Francis et al. 2010
	ROM MAM 111277	HM540704	–	–	Viet Nam	Francis et al. 2010
	CPV10-292	MH137317	–	–	Cambodia	Tu et al. 2018
	ROM MAM 110605	HM540690	–	–	Laos	Francis et al. 2010
	ROM MAM 110604	HM540705	–	–	Laos	Francis et al. 2010
	23028	MH137315	–	–	Viet Nam	Tu et al. 2018
	23036	MH137316	MH137381	MH137509	Viet Nam	Tu et al. 2018
*Kerivoulatitania*	PSUZC-MM2011.48	KY034089	–	–	Thailand	Soisook et al. 2016
	PSUZC-MM2011.15	KY034108	–	–	Thailand	Soisook et al. 2016
	VN11-0945	MH137361	–	–	Viet Nam	Tu et al. 2018
	VN11-0013	MH137349	–	–	Viet Nam	Tu et al. 2018
	23034	MH137340	MH137396	–	Viet Nam	Tu et al. 2018
	VN11-0010	MH137346	–	–	Viet Nam	Tu et al. 2018
	VN11-0025	MH137352	–	–	Viet Nam	Tu et al. 2018
	VN11-0026	MH137353	–	–	Viet Nam	Tu et al. 2018
	VN11-0027	MH137354	–	–	Viet Nam	Tu et al. 2018
	VN11-0030	MH137355	–	–	Viet Nam	Tu et al. 2018
*K.titania*	VN11-0031	MH137356	–	–	Viet Nam	Tu et al. 2018
	VN11-0944	MH137360	–	–	Viet Nam	Tu et al. 2018
	VN11-1832	MH137364	MH137402	–	Viet Nam	Tu et al. 2018
	CPV10-415	MH137343	–	–	Cambodia	Tu et al. 2018
	CPV10-363	MH137341	MH137398	MH137520	Cambodia	Tu et al. 2018
	CPV10-399	MH137342	MH137399	MH137521	Cambodia	Tu et al. 2018
	VN11-1188	MH137362	MH137401	MH137523	Viet Nam	Tu et al. 2018
	VN11-1193	MH137363	–	–	Viet Nam	Tu et al. 2018
	21942	MH137339	MH137395	MH137519	Viet Nam	Tu et al. 2018
	VN11-0002	MH137344	–	–	Viet Nam	Tu et al. 2018
	VN11-0009	MH137345	–	–	Viet Nam	Tu et al. 2018
	VN11-0011	MH137347	–	–	Viet Nam	Tu et al. 2018
	VN11-0012	MH137348	MH137400	MH137522	Viet Nam	Tu et al. 2018
	VN11-0014	MH137350	–	–	Viet Nam	Tu et al. 2018
	VN11-0018	MH137351	–	–	Viet Nam	Tu et al. 2018
	VN11-0035	MH137357	–	–	Viet Nam	Tu et al. 2018
	VN11-0040	MH137358	–	–	Viet Nam	Tu et al. 2018
	VN11-0044	MH137359	–	–	Viet Nam	Tu et al. 2018
	23041	–	MH137397	–	Vietnam	Tu et al. 2018
*Kerivoulahardwickii* s. str.	21746	MH137306	MH137373	MH137503	Indonesia	Tu et al. 2018
	VN11-1623	MH137309	MH137376	MH137505	Cambodia	Tu et al. 2018
	VN11-1593	MH137307	MH137374	MH137504	Cambodia	Tu et al. 2018
	VN11-1622	MH137308	MH137375	–	Cambodia	Tu et al. 2018
	17348	–	MH137372	–	Indonesia	Tu et al. 2018
*K.depressa*	ROM MAM 118429	HM540670	–	–	Laos	Francis et al. 2010
	AGS980322-65	HM540664	–	–	Laos	Francis et al. 2010
	ROM MAM 118186	HM540671	–	–	Laos	Francis et al. 2010
	ROM MAM 118026	HM540672	–	–	Laos	Francis et al. 2010
	ROM MAM 107721	HM540669	–	–	Viet Nam	Francis et al. 2010
	VN11-1554	MH137313	MH137379	MH137506	Cambodia	Tu et al. 2018
	VN11-1835	MH137314	MH137380	–	Vietnam	Tu et al. 2018
	ROM MAM 117982	HM540673	–	–	Laos	Francis et al. 2010
	ROM MAM 110602	HM540667	–	–	Laos	Francis et al. 2010
	ROM MAM 117971	HM540668	–	–	Laos	Francis et al. 2010
	ROM MAM 110585	HM540674	–	–	Laos	Francis et al. 2010
	CPV10-291	MH137310	MH137377	MH137507	Cambodia	Tu et al. 2018
*K.depressa*	CPV10-293	MH137311	–	–	Cambodia	Tu et al. 2018
	CPV10-409	MH137312	MH137378	MH137508	Cambodia	Tu et al. 2018
*Kerivoulakachinensis*	MAM 107718	HM540736	–	–	Viet Nam	Francis et al. 2010
	VN11-1831	MH137338	MH137394	–	Viet Nam	Tu et al. 2018
	VN11-0940	MH137337	MH137393	–	Viet Nam	Tu et al. 2018
	CPV10-416	MH137336	MH137392	MH137518	Cambodia	Tu et al. 2018
	ZMMU S-184667	GU684767	–	–	Viet Nam	Tu et al. 2018
	EDB 25747	HM540734	–	–	Laos	Francis et al. 2010
*Kerivoulafurva*	16481	MH137335	MH137391	MH137513	Nepal	Tu et al. 2018
	VN11-0050	MH137330	–	–	Viet Nam	Tu et al. 2018
	VN11-0004	MH137324	MH137387	MH137515	Viet Nam	Tu et al. 2018
	VN11-0045	MH137325	–	–	Viet Nam	Tu et al. 2018
	VN11-0046	MH137326	–	–	Viet Nam	Tu et al. 2018
	VN11-0047	MH137327	–	–	Viet Nam	Tu et al. 2018
	VN11-0048	MH137328	–	–	Viet Nam	Tu et al. 2018
	VN11-0049	MH137329	–	–	Viet Nam	Tu et al. 2018
	VN11-0943	MH137333	MH137389	MH137516	Viet Nam	Tu et al. 2018
	VN11-0937	MH137331	MH137388	–	Viet Nam	Tu et al. 2018
	VN11-0942	MH137332	–	–	Viet Nam	Tu et al. 2018
	23024	MH137322	–	–	Viet Nam	Tu et al. 2018
	23025	MH137323	MH137385	MH137514	Viet Nam	Tu et al. 2018
	VN11-1361	MH137334	MH137390	MH137517	Viet Nam	Tu et al. 2018
	2005-670	–	MH137386	–	Nepal	Tu et al. 2018

## ﻿Appendix 2

A key to the *Kerivoula* species occurring in China (both in English and Chinese)

**Table:**

1	Pelage color relatively bright. Fur orange. Wing membranes dark brown with reddish brown markings / 体色鲜艳,体毛橙黄色;翼膜深棕色,具红褐色斑块	***Kerivoulapicta*** / 彩蝠
–	Pelage color duller. Fur dark gray, brown, or blackish gray / 体色较暗淡,体毛暗灰色、棕色或黑灰色	**2**
2	Size larger, forearm length more than 40 mm. Greatest length of skull more than 16 mm, greatest breadth of braincase more than 8 mm / 体型较大,前臂长超过 40 mm; 颅全长大于 16 mm, 脑颅宽大于 8 mm	***Kerivoulakachinensis*** / 克钦彩蝠
–	Size smaller, forearm length less than 40 mm. Greatest length of skull less than 16 mm, greatest breadth of braincase less than 8 mm / 体型较小,前臂长不及40 mm;颅全长小于16 mm,脑颅宽小于8 mm	**3**
3	Tibia length usually more than 18.5 mm. Greatest length of skull 14.5–16.0 mm, braincase height usually more than 5.3 mm / 胫骨长常大于 18.5 mm; 颅全长 14.5–16 mm, 颅高常大于 5.3 mm	***Kerivoulatitania*** / 泰坦尼亚彩蝠
–	Tibia length less than or equal to 18.5 mm. Greatest length of skull less than 14.5 mm, braincase height usually less than 5.3 mm / 胫骨长小于或等于18.5 mm; 颅全长小于14.5 mm, 颅高不及5.3 mm	**4**
4	Greatest width across the outer edges of the third upper molars more than 5.3 mm, greatest length of mandible more than 9.8 mm. Dorsal pelage dark brown to blackish gray, broadly uniform in colour from bases to tips / 第三上臼齿宽大于5.3 mm,下颌长大于9.8 mm;背毛深棕色到黑灰色,毛基到毛尖颜色 大致相同	***Kerivoulafurva*** / 暗褐彩蝠
–	Greatest width across the outer edges of the third upper molars less than 5.3 mm, greatest length of mandible less than 9.8 mm. Dorsal pelage buff brown to dark brown, its base and tip significantly different / 第三上臼齿宽小于5.3 mm,下颌长小于9.8 mm;背毛浅棕色到深棕色,毛基与毛尖颜色明显不同	**5**
5	Braincase slightly flattened, braincase height more than 4.7 mm, more than 1/3 of greatest length of skull. Dorsal pelage buff brown to dark brown, ventral pelage dark brown / 脑颅略为扁平,颅高大于4.7 mm,超过颅全长1/3;背毛浅棕色到深棕色,腹毛深棕色	***Kerivouladepressa*** / 平颅彩蝠
–	Braincase flatter, braincase height usually less than 4.7 mm, less than 1/3 of greatest length of skull. Dorsal pelage dark brown to golden brown, ventral pelage golden brown / 脑颅更扁平,颅高多小于4.7 mm,不及颅全长1/3;背毛深棕色到金棕色,腹毛金棕色	***Kerivouladongduongana*** / 印支彩蝠
